# Human Color Perception: The Impact of Color Perception on Fine-Grained Emotion Prediction in Movie and Television Videos

**DOI:** 10.3390/s24237770

**Published:** 2024-12-04

**Authors:** Shuang Wang, Jinyu Liu, Yiming Feng, Xiaomeng Ren, Huibo Hu, Mengfei Zhang, Zhibin Su

**Affiliations:** 1 State Key Laboratory of Media Convergence and Communication, Communication University of China, Beijing 100024, China; wangshuang@cuc.edu.cn; 2 Key Laboratory of Acoustic Visual Technology and Intelligent Control System, Communication University of China, Ministry of Culture and Tourism, Beijing 100024, China; ljy_123@cuc.edu.cn (J.L.); fyming8888@163.com (Y.F.); rxm_cuc@163.com (X.R.); hhb_cuc@163.com (H.H.); 202120081002009@cuc.edu.cn (M.Z.); 3 Beijing Key Laboratory of Modern Entertainment Technology, Communication University of China, Beijing 100024, China

**Keywords:** subjective evaluation experiment, random forest

## Abstract

This article investigates the impact of visual color perception on fine-grained emotion prediction in videos, analyzing the contribution of color perception features in fine-grained emotion prediction. A total of 20 subjects were involved in this experiment. First, 10 subjects conducted a fine-grained emotional subjective evaluation experiment on 50 video clips. Then, another 10 subjects conducted a subjective evaluation experiment of color perception annotation for these 50 video clips. On this basis, the correlation and mechanism between color perceptual features and fine-grained emotions were analyzed. Finally, a fine-grained emotion prediction model was established based on the combination of objective features and color perceptual features. It was also observed that, compared to using only objective features, incorporating perceptual features enhanced the model’s accuracy. This article also compared the importance of different perceptual features for each emotion and explained the mechanism between color perception and fine-grained emotions. By selecting the top 24 important features to predict emotions, the association between perceptual features and emotions was more effectively captured.

## 1. Introduction

Researchers have shown that different color stimuli can evoke varied perceptual responses [1]. For instance, red can lead to a sense of warmth, while blue is often associated with coldness [2,3]. The influence of color extends beyond the level of perception to deeper psychological effects, such as emotional impact [4]. Different color perceptions can also induce diverse emotions and feelings [5], with warm colors associated with happiness and cool colors with sadness. In real life, images are rich with colors and emotions. People’s emotional cognition of images is influenced by color perception. Studies have also substantiated the role of color perception in emotion. Mitsuhiko Hanada et al. [5] proposed a correspondence between the color wheel and the emotional/affective circular model, and constructed an eight-bit bipolar chart through experiments to test the hypothesis of emotion–color associations. R. Gong et al. [6] empirically verified a certain correlation between color emotion and color preference, and through a factor analysis, demonstrated that color emotion does not exist in isolation. Wang et al. [1] extracted nine multicolor perceptual features based on individuals’ perceptual responses to the simultaneous influence of multiple colors, and further proposed MCEG-NET by integrating color fundamental properties, multicolor physical features, and multicolor perception features [7].

However, the colors observed in daily life do not appear in the form of single colors or simple color combinations, but rather in the dynamic and realistic representation of objects and scenes. These color dynamics have a substantial impact on emotional perception, influencing the moods of observers and eliciting specific emotional responses. As a result, it is essential to comprehend the correlation between color and emotion in videos in order to more accurately mimic emotional responses. Zhang et al. [8] identified emotions based on the physical elements in videos, analyzing the physical elements in the two-dimensional emotion map by extracting video physical elements, and summarizing the relationship between the two-dimensional emotion space and physical elements. Cakmak et al. [9] established a robust color–emotion baseline dictionary, mapping specific colors into the spectrum of human emotions, revealing the complex ways in which visual tones impact audience emotions. Anshu et al. [10] asserted that emotions influence thoughts, feelings, and actions, with color serving as the primary source of these sensations. Accordingly, based on users’ emotions, they proposed an emotion-driven movie recommendation system rooted in color psychology.

The interaction between color and emotion involves complex mechanisms that go beyond a simple mapping relationship. The development of machine learning has provided new solutions for modeling color and emotion. Wȩdołowska et al. [11] developed a machine learning algorithm to classify emotions based on the colors presented in images, predicting emotions based on the colors presented in images and video excerpts. Jonauskaite et al. [12] proposed a sophisticated emotion data analysis method based on machine learning.

Emotional expressions rely on contextual connections [13], and understanding emotions when viewed through the lens of photography and film is more complex than a single image. Current research using simple physical elements and traditional two-dimensional emotion spaces does not fully accommodate diverse emotions, and as such, has some limitations. Furthermore, with the development of technology and society, people’s aesthetic demands regarding color and emotion are increasing. Various applications with emotional functionalities also need to be optimized. This requires us to consider the application scenarios of color and the diversity of emotions in reality. It is necessary to study the mechanism of color perception on emotion at the level of video while improving the accuracy of emotion description.

Individuals from different backgrounds perceive colors and emotions in drastically different ways [9], with a greater wealth of social experiences leading to increased complexity in personal associations [14]. This fact complicates the issues. This study aims to explore the universal association between color features and emotions, thus avoiding the influence of cultural differences and personal factors on emotion prediction.

**Figure 1:**
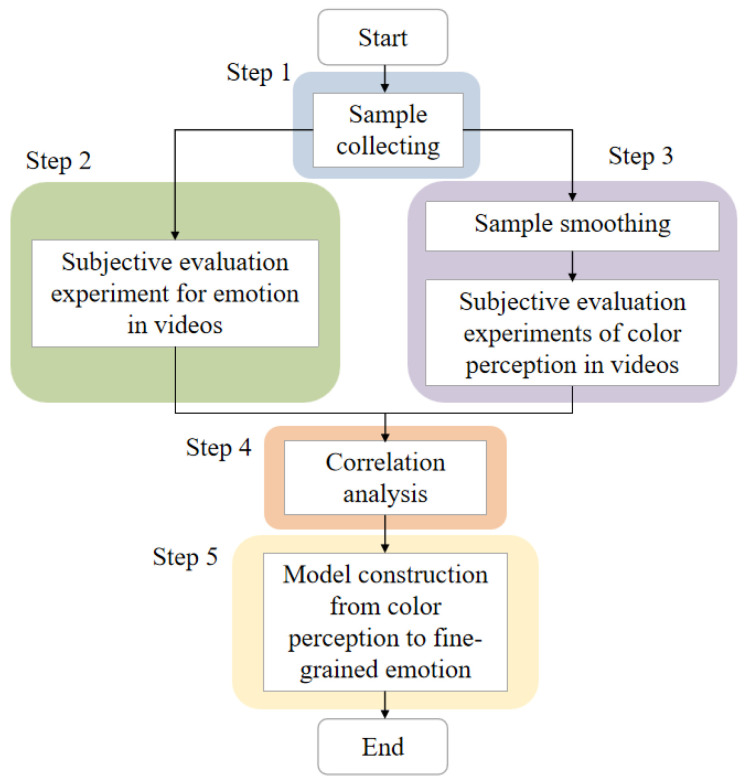
Research framework for correlation of color perception with emotion in videos.

In general, the research framework is shown in Figure 1. In Step 1, film and video clips are selected as the source of samples (see Section 2). In step 2, a subjective evaluation experiment is conducted for the fine-grained emotion space on the muti-scale approach (see Section 2). In step 3, a subjective evaluation experiment is conducted for the color perceptual features based on the multi-scale method (see Section 3). In step 4, the correlation between fine-grained emotion words and perceptual features is analyzed (see Section 4). In step 5, a random forest algorithm is utilized to construct an emotion prediction model based on perceptual features (see Section 4).

**Figure 2:**
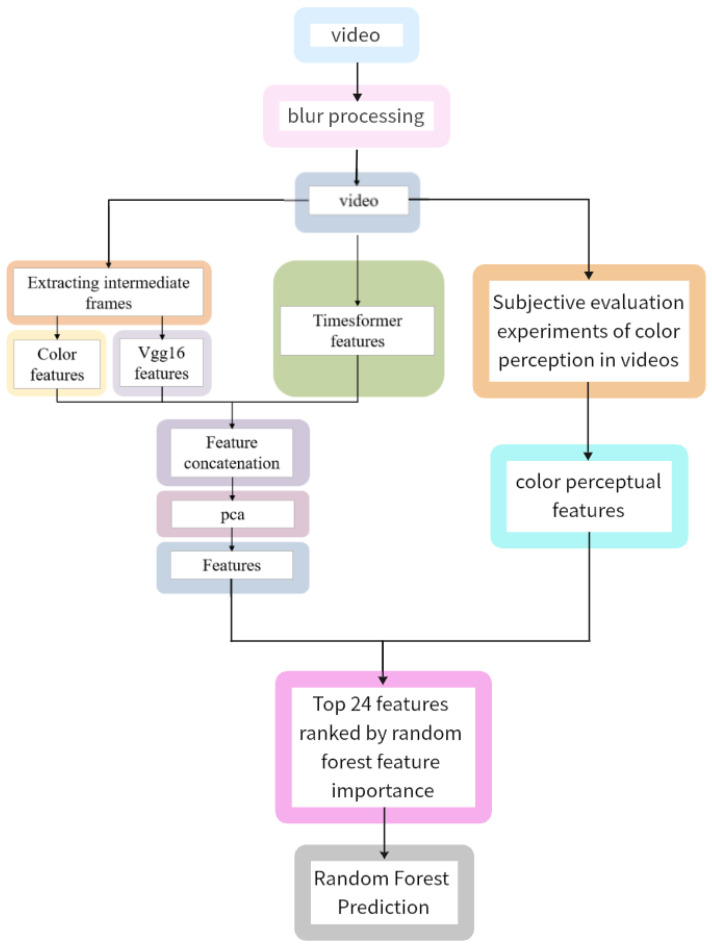
Framework of proposed emotion prediction algorithm.

In conclusion, the primary contributions of this investigation are as follows:

- Revealing the association between color perception features and emotions: We systematically analyzed the relationship between color perception features and fine-grained emotions, addressing the gap in previous studies regarding the analysis of subjective color perception and its emotional associations.
- Exploring the mechanism of color perception features: Through experiments, we demonstrated the significant contribution of subjective color perception features to emotion prediction, uncovering their core role in emotion elicitation and expression, and providing a theoretical basis for deeper emotion modeling.
- Proposing an emotion prediction framework combining objective features and color perception features: We innovatively integrated subjective color perception features with objective features to construct a comprehensive model that enhances the performance of fine-grained emotion prediction, as illustrated in Figure 2. The effectiveness of this approach was validated through experiments.

These results not only offer new insights for optimizing emotion prediction models, but also have significant practical implications for visual media design, user experience enhancement, and the development of emotional interaction systems.

## 2. Subjective Evaluation Experiments for Video Emotion

### 2.1. Experimental Design

#### 2.1.1. Subjects

Ten subjects participated in the experiment. All of them were graduate students around 22 years old. No subjects were majoring in vision science. The male-to-female ratio was close to 1:1. Each subject was asked to take the Ishihara Color Blindness Test [15] to ensure that their color vision was normal.

#### 2.1.2. Materials

**Figure 3:**
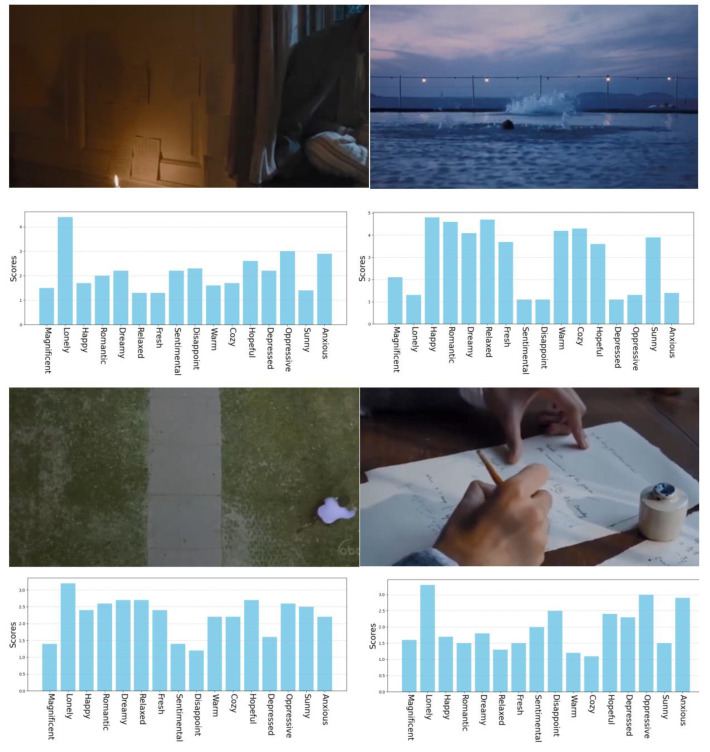
Film and television scene video dataset.

Film and television scene videos contain rich emotional semantic information. Different from the content taken by ordinary users, the emotions in film and television scene videos are presented by professional teams after careful creation, which has originality, higher aesthetic value, and research significance. Fifty movie and television clips of 3 to 6 s were selected as samples for this experiment with a resolution of 1280 × 720 dpi, as shown in Figure 3. To avoid the influence of music factors on emotions, the video clips used in this experiment were mute videos. In addition, 20% replicate samples (10 videos) were designed for each experiment to allow for repeated tests during the data analysis.

#### 2.1.3. Fine-Grained Emotion Space

The 16-dimensional fine-grained emotion space obtained from the pre-experiment is used to evaluate the emotion values of the samples. The process of pre-experimentation and calculation to establish the fine-grained emotion space is as follows:

- First remove words that are not related to human cognition from the adjective vocabulary collected at the beginning, usually adjectives used to describe people’s character, morality, or environmental things. After that, the definition of adjectives is clarified with the help of a modern Chinese dictionary. Next, the words with similar meanings are grouped and selected, retaining representative words to form the initial emotion vocabulary set $w=\{w_{1},w_{2},...,w_{m}\}$.
- The set of sentiment words is determined by subjective evaluation experiments. A total of 30 undergraduates and postgraduates from the Communication University of China were randomly selected to view the image library in random order, and the emotional intensity of each image was evaluated for each emotion word. This process resulted in the initial scoring matrix R:

(1) $$R=\left[\begin{array}{ccc} r_{11} & \cdots & r_{1m}\\ \vdots & \ddots & \vdots \\ r_{n1} & \cdots & r_{nm} \end{array}\right]$$

where $r_{ij}$ represents the score assigned to the i image for the j emotion word. The scoring matrix R was standardized as follows:

(2) $${r\prime }_{ij}=\frac{r_{ij}-\mu_{j}}{\sigma_{j}}$$

(3) $$R\prime =\left[\begin{array}{ccc} {r\prime }_{11} & \cdots & {r\prime }_{1m}\\ \vdots & \ddots & \vdots \\ {r\prime }_{n1} & \cdots & {r\prime }_{nm} \end{array}\right]$$

where $\mu_{j}$ and $\sigma_{j}$ are the mean and standard deviation of the j column, respectively.

c. A smaller set of emotion words was selected through a cluster analysis. First, the distance between emotion words was calculated based on the scoring matrix:

(4) $$d(w_{i},w_{j})=\sqrt{\sum_{k=1}^{n}{\left({r\prime }_{ki}-{r\prime }_{kj}\right)}^{2}}$$

This yielded the distance matrix D:

(5) $$D=\left[\begin{array}{ccc} 0 & \cdots & r_{1m}\\ \vdots & \ddots & \vdots \\ d_{m1} & \cdots & 0 \end{array}\right]$$

The distance matrix D was then input into a hierarchical clustering algorithm to form groups: ${Group}_{i}=\{w_{i1},w_{i2},...\}$. For each group, the word with the smallest average distance to all other words in the group was selected as the representative word:

(6) $$w_{center}=arg\underset{w_{i}\in \mathrm{Group}}{\min}\frac{1}{\left|Group\right|}\sum_{w_{i}\in Group}d(w_{i},w_{j})$$

Finally, the 16-dimensional fine-grained emotion space was constructed.

The 16-dimensional fine-grained emotion space used in the experiment to evaluate the sentiment of the sample set of words is shown in Table 1.

#### 2.1.4. Experimental Conditions

**Figure 4:**
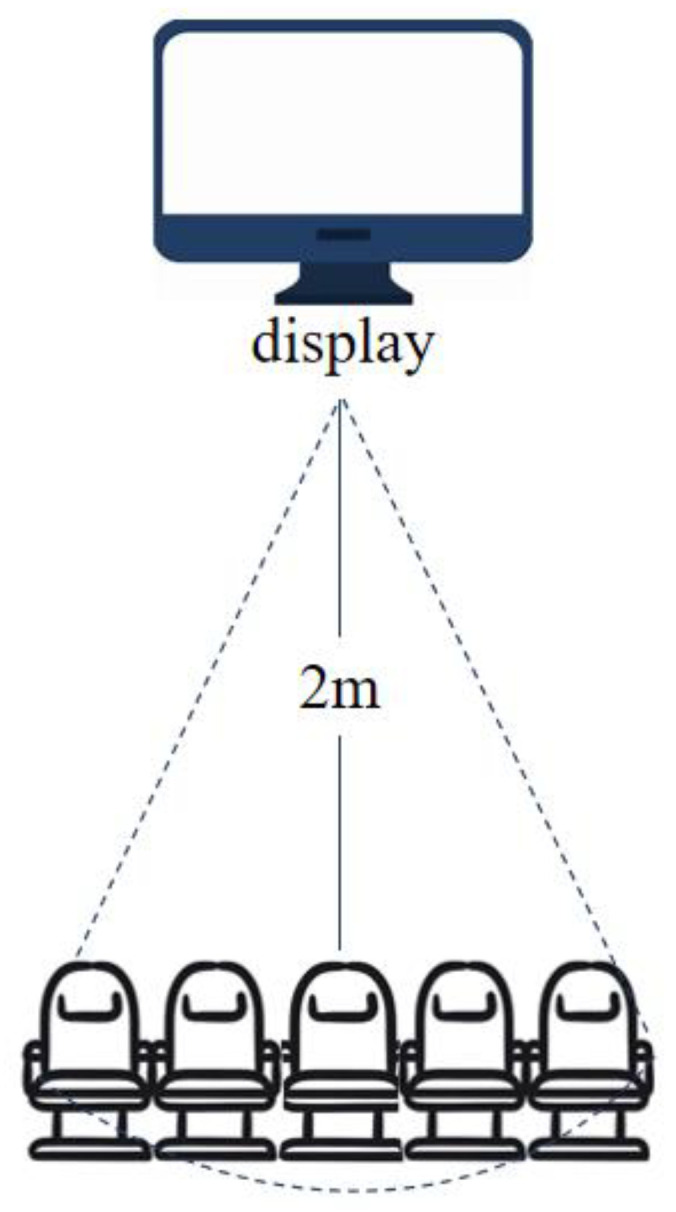
The seat arrangement.

To avoid the interference of noise to the emotional evaluation, the experiment was carried out in an underground standard listening room with an area of 5.37 m × 6 m, where the background noise was kept below 30 dB. The samples were presented randomly on a 75-inch SONY KD-75X9400D HD display screen. According to the Methodologies for the Subjective Assessment of the Quality of Television Pictures [16], environmental illuminance on the screen (incident light from the environment falling on the screen, should be measured perpendicularly to the screen) was set to 200 lx (an illumination unit, representing the luminous flux received on the display per unit area). There are a total of 10 subjects in the experiment, divided into 2 groups, with 5 subjects in each group. As shown in Figure 4, the seats were arranged so that the distance and angle of each subject were as comfortable as possible.

#### 2.1.5. Experimental Procedure

The subjects were asked to score all the samples on a 5-level scale of descriptive variables after fully watching the videos, and each subject filled out the questionnaire at most once. Please ensure that each subject completes the experiment voluntarily after fully understanding the content of the experiment.

Taking “lonely” as an example, 1 means definitely not (not feeling lonely at all), 2 means somewhat (seeming to feel lonely, but not very sure), 3 means somewhat but not very much (sure I feel lonely, but not very much), 4 means strongly (I feel lonely obviously, but strongly), and 5 means very strong (I can clearly feel lonely, and very strongly).

### 2.2. Reliability Analysis

A repeated test and Cronbach’s alpha were adopted to evaluate the reliability of the experimental data. A repeated test is used to measure the reliability of each subject. In the case of the five-level scale, if the probability that the same subject’s rating difference is greater than or equal to two levels in two repeated measures of the same video is less than 15%, the subject is considered reliable. Cronbach’s alpha is used to measure the internal consistency of the evaluation results (Intraclass Correlation Coefficient, ICC); usually above 0.7 is considered to be more reliable [17]. All subjects were found to be reliable by manual inspection. On this basis, Cronbach’s alpha was adopted to validate the ICC of the experimental data:

(7) $$\alpha =\frac{K}{K-1}\left(1-\frac{\sum_{i=1}^{K}\sigma_{i}^{2}}{\sigma_{X}^{2}}\right)$$

where K represents the number of stimuli, $\sigma_{i}^{2}$ represents the score variance of all the subjects on the *i*th measurement item, and $\sigma_{X}^{2}$ represents the total variance of the total scores obtained by all the subjects.

As shown in Table 2, all the scales have very good ICC, the reliability coefficients are all greater than 0.7, and the analysis results meet the reliability requirements, so that the experimental data are reliable.

## 3. Subjective Evaluation Experiments for Color Perception

### 3.1. Experimental Design

#### 3.1.1. Subjects

Ten subjects participated in this experiment (there was no repetition of the subjects in the subjective emotion experiment). All the subjects were graduate students around 22 years old, and the male-to-female ratio was close to 1:1. All the subjects were healthy without eye diseases, and their majors were unrelated to visual science and aesthetics. Before the official start of the experiment, each subject was asked to take the Ishihara Color Blindness Test to ensure that their color vision [15] was normal.

#### 3.1.2. Materials

**Figure 5:**
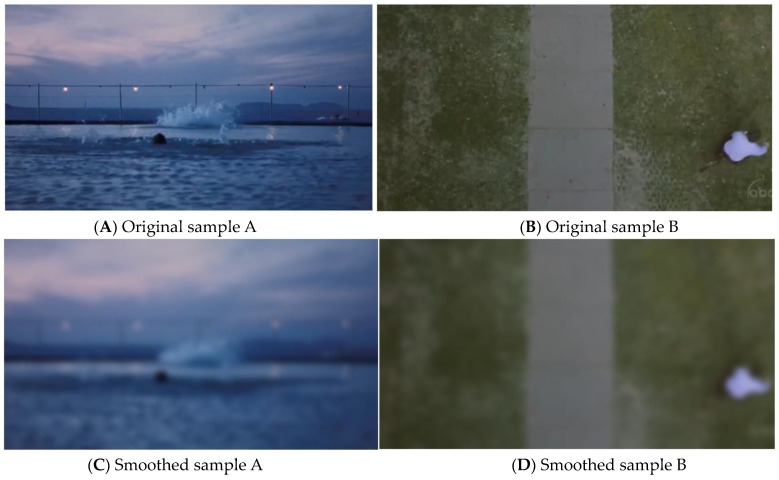
The differences between the original sample and the smoothed one.

The 50 film and television clips of 3 to 6 s were still selected as the samples of this experiment. The samples contain not only color information but also edge information. People can recognize objects through edge information, so they can process more semantic information, which may affect the perceptual evaluation of color [1]. Therefore, in the subjective evaluation experiment for color perception, in order to avoid the information interference of picture semantics, this experiment uses a 50-pixel circular template to perform mean filtering on the 50 videos, which blurs the semantic information on the premise of ensuring the consistency of color. Figure 5 shows the difference between the original and smoothed samples. Figure 5A shows the original sample and Figure 5B shows the smoothed sample. The semantic information in the original samples, such as expressions and words, is blurred. Similarly, Figure 5C shows the original sample and Figure 5D shows the smoothed sample.

During the experiment, similar to the video emotion subjective evaluation experiment, 20% repeated samples (10 videos) were designed for each experiment, so that the repeated test could be carried out during the data analysis.

On this basis, as shown in Table 3, nine perceptual descriptive words, namely “dynamic/static”, “gaudy/plain”, “cool/warm”, “light/dark”, “light/heavy”, “soft/hard”, “transparent/turbid”, “far/near”, and “swell/shrink”, were adopted for 10 subjects to evaluate 50 video clips of film and TV scenes.

#### 3.1.3. Experimental Conditions

The experimental condition was consistent with that described in Section 2.1.4, which was carried out in the underground standard listening room of 5.37 m × 6 m.

#### 3.1.4. Experimental Procedure

After fully watching the video, each subject scored each sample on a 5-level scale of descriptive variables, and each subject filled out the questionnaire at most once. It was ensured that each subject completed the experiment voluntarily after fully understanding the content of the experiment.

Taking “cold/warm” as an example, 1 means cold, 2 means somewhat cold, 3 means no feeling, 4 means somewhat warm, and 5 means warm.

### 3.2. Reliability Analysis

This experiment also used repeated tests and Cronbach’s alpha for the reliability analysis in a manner consistent with that described in Section 2.2. After the inspection and analysis, all subjects were valid subjects, and on this basis, Cronbach’s coefficient was applied to verify the ICC of the experimental scribe. Table 4 shows that the reliability coefficients are all greater than 0.7, which meets the reliability requirements; that is, the experimental data are reliable [17].

## 4. Results and Analysis

### 4.1. Correlation Analysis Between Color Perception and Emotion

This section analyzes the correlation between perceived quantity and sentiment words based on the experimental results. In order to eliminate the dimensional differences in experimental data, this paper first normalizes the experimental data. The normalization formula is shown in Equation (8):

(8) $$x_{i}=\frac{x_{i}-\min\left\{x\right\}}{\max\left\{x\right\}-\min\left\{x\right\}}$$

where $x_{i}$ is the *i*th sample; $\max\left\{x\right\}$ and $\max\left\{y\right\}$ are the maximum observations of $\left\{x_{1},x_{2},\ldots ,x_{n}\right\}$ and $\left\{y_{1},y_{2},\ldots ,y_{n}\right\}$; $\min\left\{x\right\}$ and $\min\left\{y\right\}$ are the minimum observations of $\left\{x_{1},x_{2},\ldots ,x_{n}\right\}$ and $\left\{y_{1},y_{2},\ldots ,y_{n}\right\}$.

The Pearson correlation coefficient was adopted to evaluate the correlation

(9) $$r_{xy}=\frac{\sum_{i=1}^{n}\left(x_{i}-\bar{X}\right)\left(y_{i}-\bar{Y}\right)}{\sqrt{\sum_{i=1}^{n}{\left(x_{i}-\bar{X}\right)}^{2}}\sqrt{\sum_{i=1}^{n}{\left(y_{i}-\bar{Y}\right)}^{2}}}$$

where n is the number of samples; $x_{i}$ and $y_{i}$ are the *i*th observations of $\left\{x_{1},x_{2},\ldots ,x_{n}\right\}$ and $\left\{y_{1},y_{2},\ldots ,y_{n}\right\}$.

In this study, Pearson’s correlation coefficients between color perception features (including “dynamic/static”, “gaudy/plain”, “cool/warm”, “light/dark”, “light/heavy”, “soft/hard”, “transparent/turbid”, “far/near”, and “swell/shrink”) and each dimension of the fine-grained emotions are as shown in Table 5.

To simplify the calculation, the absolute value of the correlation coefficient was analyzed as a statistical value. According to Table 5, there are 18 groups of perceptual amount–emotion words with correlation coefficients greater than 0.3, which are “dynamic/static”–“magnificent”, “gaudy/plain”–“magnificent”, “gaudy/plain”–“lonely”, “light/dark”–“lonely”, “light/heavy”–“fresh”, “light/heavy”–“oppressive”, “light/heavy”–“anxious”, “soft/hard”–“romantic”, “soft/hard”–“fresh”, “soft/hard”–“warm”, “soft/hard”–“cozy”, “transparent–turbid”–“relaxed”, “transparent–turbid”–“fresh”, “far/near”–“sentimental”, “far/near”–“disappointed”, “soft/hard”–“hopeful”, “swell/shrink”–“relaxed”, and “swell/shrink”–“fresh”. Among them, “magnificent” and “gaudy/plain” have the strongest correlation with a correlation coefficient of 0.785. The evaluation results show that there is a correspondence between color perception and sentiment words.

### 4.2. Emotion Value Prediction Model Construction

**Figure 6:**
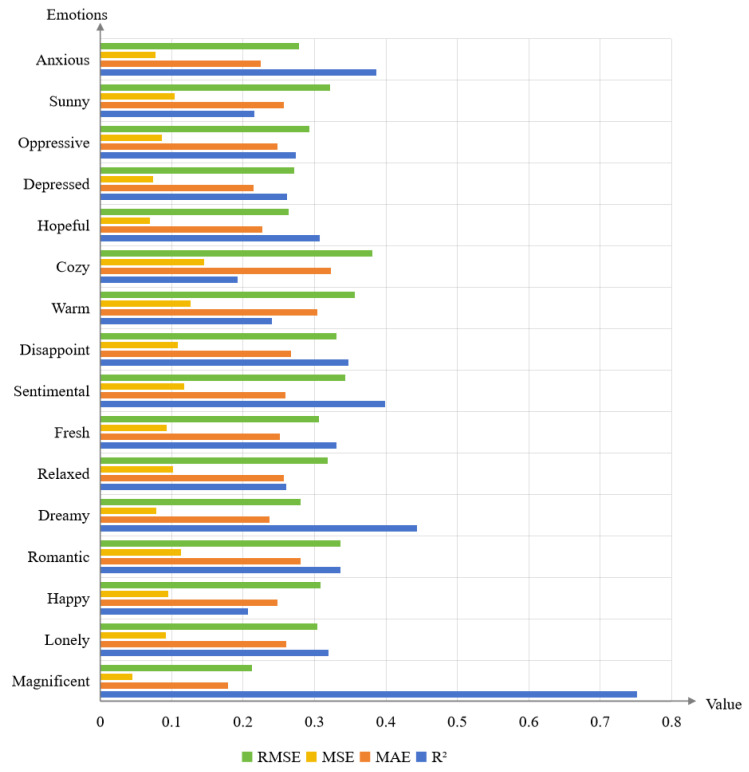
The 5-fold cross-validation result of the MLR model.

This study constructs a mathematical model using a multiple linear regression algorithm to capture the relationship between salient color perception features and fine-grained emotions. Mean Squared Error (MSE), Mean Absolute Error (MAE), and Root Mean Squared Error (RMSE) are employed as evaluation metrics for the experiments, as shown in Equations (10)–(12). The model’s accuracy is evaluated using 5-fold cross-validation, where the dataset is divided into five subsets and four of them are used as training sets to predict the remaining subset. The mathematical model of linear regression is presented in Equations (13)–(28), and the prediction results are summarized in Figure 6.

(10) $$SE=\frac{1}{m}\sum_{i=1}^{m}{\left(y_{i}-\hat{y_{i}}\right)}^{2}$$

(11) $$MAE=\frac{1}{m}\sum_{i=1}^{m}\left|y_{i}-\hat{y_{i}}\right|$$

(12) $$RMSE=\sqrt{\frac{1}{m}\sum_{i=1}^{m}{\left(y_{i}-\hat{y_{i}}\right)}^{2}}$$

From Figure 6, it can be observed that the highest R^2^ value for “magnificent” is 0.751, as shown in Equation (4). Negative correlations are found between “gaudy/plain” and “magnificent”, while positive correlations exist between “transparent/turbid”, “far/near”, “swell/shrink”, and “magnificent”. Moreover, the study reveals that modeling emotion prediction with selected significant perceptual features, determined through the significance analysis, performs worse compared to using all perceptual features. This could be attributed to the fact that color perceptual features influence each other and involve complex nonlinear relationships with fine-grained emotions.

(13) Magnificent=0.217DS−0.846GP−0.094CW−0.126LD−0.347LH+0.237SH−0.731TT−0.293FN+0.484SS+1.435

(14) Lonely=0.173DS+0.221GP−0.024CW+0.304LD+0.290LH+0.259SH−0.378TT−0.367FN−0.738SS+0.375

(15) Happy=−0.033DS−0.017GP+0.105CW−0.258LD−0.240LH−0.328SH+0.063TT+0.125FN+0.885SS+1.398

(16) Romantic=0.387DS−0.291GP+0.143CW+0.004LD+0.091LH−0.495SH−0.476TT−0.092FN+1.009SS+2.422

(17) Dreamy=0.227DS−0.423GP+0.112CW+0.073LD−0.254LH−0.155SH−0.317TT+0.844FN+3.367SS+3.367

(18) Relaxed=−0.025DS+0.038GP+0.060CW−0.174LD−0.530LH−0.142SH−0.052TT+0.098FN+1.078SS+1.258

(19) Fresh=0.164DS−0.009GP+0.112CW−0.164LD−0.461LH−0.133SH−0.149TT−0.074FN+0.999SS+1.599

(20) Sentimental=0.422DS−0.147GP+0.116CW+0.179LD+1.027LH−0.106SH−0.653TT−0.730FN−0.591SS+4.781

(21) Disappoint=0.237DS+0.058GP+0.180CW+0.207LD+0.630LH+0.092SH−0.662TT−0.667FN−0.523SS+4.339

(22) Warm=0.199DS−0.062GP+0.228CW−0.188LD−0.044LH−0.391SH−0.036TT−0.146FN−0.992SS−0.251

(23) Cozy=0.214DS−0.034GP+0.152CW−0.197LD+0.040LH−0.463SH−0.101TT−0.026FN+0.699SS+1.861

(24) Hopeful=−0.168DS−0.096GP−0.027CW−0.159LD−0.446LH−0.091SH+0.461TT+0.479FN+0.699SS+0.445

(25) Depressed=0.188DS+0.047GP+0.085CW+0.195LD+0.669LH+0.060SH−0.400TT−0.473FN−0.548SS+3.057

(26) Oppressive=0.060DS+0.031GP+0.039CW+0.194LD+0.660LH+0.137SH−0.244TT−0.448FN−0.948SS+4.400

(27) Sunny=−0.169DS+0.039GP+0.087CW−0.292LD−0.435LH−0.201SH+0.340TT+0.190FN+0.921SS+0.557

(28) Anxious=0.287DS+0.091GP+0.062CW+0.153LD+1.116LH−0.105SH−0.636T−0.548FN−0.853SS+4.489

In order to further research the complex relationship between color perceptual features and emotions, the random forest algorithm was adopted to construct the sentiment prediction model based on perceived quantity. Random forest [18] is a widely used ensemble learning algorithm, which can obtain the ranking of the contribution of each feature to the model, so as to facilitate a further analysis of the data, including selecting important features and optimizing model parameters. In this model, the samples and features are randomly sampled to construct a number of uncorrelated decision trees, in which each tree can make a prediction according to the extracted data, and each tree in the forest is processed in parallel to perform the above operations. Finally, the results of all decision trees are integrated to obtain the regression prediction results of the whole random forest.

**Figure 7:**
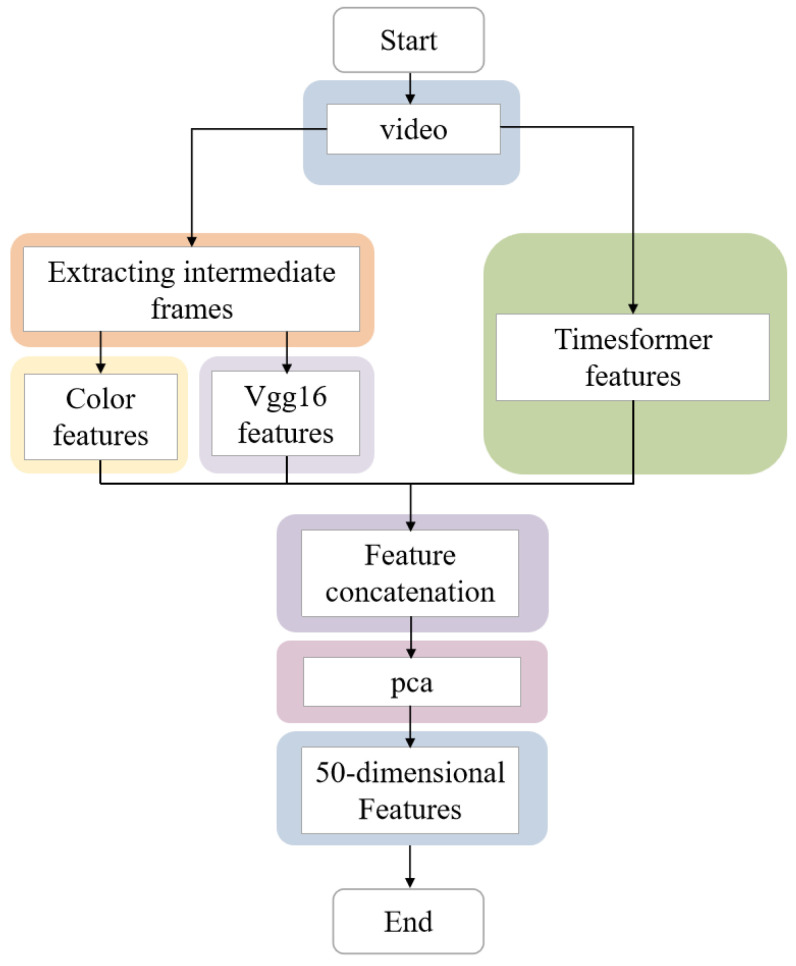
Flowchart of feature extraction.

During the video feature extraction phases, as shown in Figure 7, three types of video features were extracted, which are color features, Vgg16 features, and Timesformer features.

Firstly, for video feature extraction, this study extracts the middle frames of the videos. Based on this, MATLAB(version 2020b) was used to extract 160-dimensional color features.

Secondly, the pre-trained Vgg16 model is used to extract deep features from the middle frames of the videos. Vgg16 is a classic convolutional neural network model widely applied in image classification and object detection fields. It consists of convolutional layers, pooling layers, and fully connected layers, possessing advantages such as good expressiveness, ease of training, and strong robustness. In this study, in addition to extracting the middle frames of the videos, the Vgg16 model is employed to extract 4096-dimensional deep features from them.

Lastly, the pre-trained Timesformer model is utilized to extract deep features from the videos. The Timesformer model is a Transformer-based architecture used for video classification. The Transformer model is a deep neural network model based on self-attention mechanisms, commonly used for processing sequential data, such as language sequences. Timesformer extends the concept of Vision Transformer to the video domain, modeling the spatial and temporal information in video frames based on self-attention mechanisms, capable of capturing long-range dependencies. In this study, ffmpeg is employed to extract keyframes at a rate of one frame per second. After keyframe extraction, the pre-trained Timesformer model is used to learn spatiotemporal features from a series of frame-level patches, resulting in 400-dimensional frame-level features.

These various features are directly merged by concatenating them column-wise. The principal component analysis (PCA) method is then utilized to reduce the dimensions of the features to 50 dimensions, achieving an explanatory rate of over 97%, ensuring the retention of over 97% of the original information. These reduced features serve as the physical characteristics of the video. Subsequently, the 50-dimensional physical characteristics of the video, along with the 9-dimensional perceptual characteristics, collectively form the video features, which are used as input features for the model.

In this study, 80% of the samples are selected as the training set. To explore the impact of video blurring on emotion prediction, this study separately extracts the physical features of the original videos and the videos blurred by mean filtering, both combined with the 9-dimensional perceptual features for emotion prediction. As shown in Table 6, compared to using the features of the original videos, the physical features of the blurred videos can reduce prediction errors and improve the model’s predictive performance.

To investigate the complex nonlinear relationship between features and emotions, we further compared the random forest algorithm with two other algorithms: the support vector regression (SVR) algorithm and the multi-layer perceptron (MLP) algorithm. As shown in Table 7, the random forest algorithm performed the best, followed by SVR, and MLP performed the worst. This is because there exists a complex nonlinear relationship between video features and fine-grained emotions. The random forest algorithm exhibits strong adaptability to complex relationships, effectively capturing the nonlinear relationships between features by integrating multiple decision trees. In comparison, SVR and MLP show more limited performance in handling complex nonlinear relationships.

**Figure 8:**
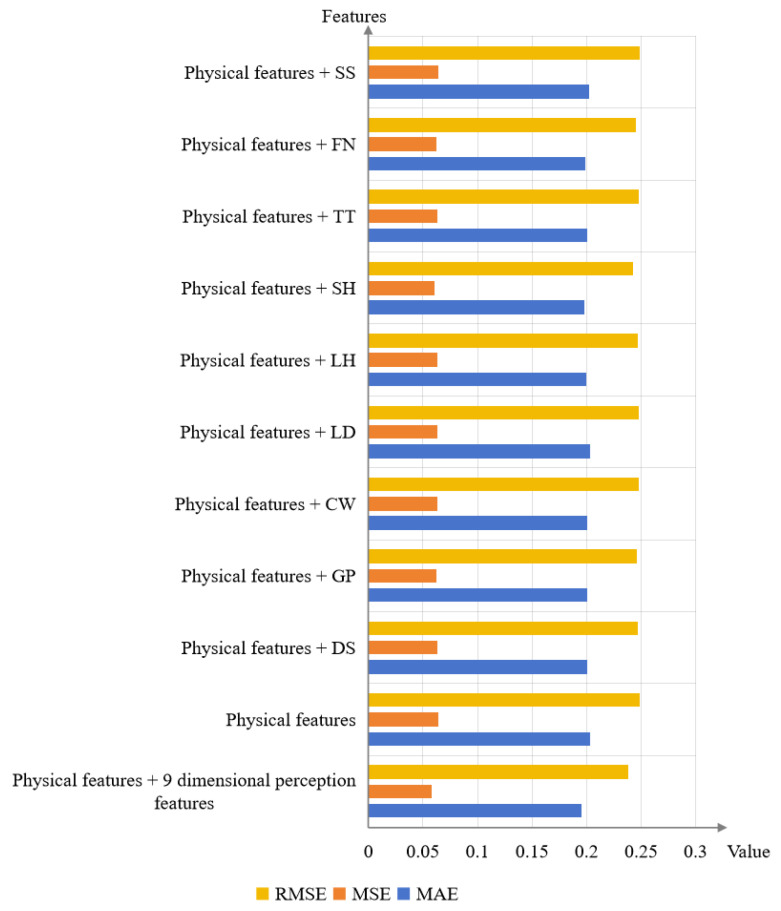
The prediction performance of different input features on the model from visual features to fine-grained emotions.

Meanwhile, Table 8 and Figure 8 show the influence of each feature combination on the emotion value prediction results. By comparison, the MEA, MSE, and RMSE are the smallest when the physical features and all the perceptual features are used as input, which proves that the nine perceptual features of color affect each other and act together on emotion. Moreover, the prediction performance decreases significantly without perceptual features, and the average prediction performance of all sentiment words is worse when only one-dimensional perceptual features are input than when all perceptual features are used.

However, for a certain emotion word, only inputting physical features with one-dimensional perceptual features is better than using physical features with all perceptual features. For example, for the emotion word ‘magnificent’, when using only physical features and the perceptual feature ‘gaudy/plain’ as input, the MAE, MSE, and RMSE are 0.195, 0.056, and 0.237. In contrast, when using physical features with all perceptual features as input, the MAE, MSE, and RMSE increase to 0.218, 0.066, and 0.257, respectively. This is because there may be some correlation between color perception features and certain emotions, leading to different performance on different emotion words using different feature combinations. Furthermore, for the emotion word “magnificent”, the perceptual feature “gaudy/plain” has the largest correlation coefficient, with Pearson’s correlation coefficient of 0.785, echoing the prediction result of random forest.

### 4.3. Feature Analysis

In order to further verify the action mechanism of color perceptual features on emotions and enhance the explainability of perceptual features and emotions, we calculated the importance of each color perceptual quantity for each emotion. Random forest’s feature importance ranking can be used to implement feature selection: based on the ranking of feature importance, features with a significant impact on the model’s predictive results can be retained, thus reducing feature dimensionality and computational complexity. By accumulating the contribution of each feature to node partitioning across multiple decision trees, the importance of individual features can be calculated. Therefore, this study used random forest importance ranking to compute the importance scores of the features based on 16 emotion words, thereby ranking the 59-dimensional features by importance. Table 9 shows the top-ranked perceptual features among nine perceptual features and the importance value of the perceptual feature corresponding to each emotion word.

Table 9 shows that the random forest importance analysis results are generally consistent with the results of perceived quantity and sentiment word correlation in Section 4.1.

The contribution of different perceptual quantities to different emotion words is quite different. The perceptual quantity “gaudy/plain” has a major contribution to the prediction of the emotion “magnificent” and a large contribution to the prediction of “lonely”. This could mean that the magnificence of the color plays an important role in expressing “magnificent” and “lonely”, possibly related to the hue, saturation, etc. Combined with Table 5, the correlation between “magnificent” and “gaudy/plain” is -0.785, which is negative, indicating that “gaudy” plays a positive role in stimulating “magnificent”, while the correlation between “lonely” and “gaudy/plain” is 0.367. This means that “plain” inspires “lonely”. Similarly, “soft/hard” contributes more to the prediction of fine-grained emotions such as “happy”, “romantic”, “dreamy”, “warm”, “cozy”, and “oppressive”. “Light/heavy” contributes more to the prediction of fine-grained emotions such as “relaxed”, “fresh”, “sunny”, and “anxious”. “Far/near” contributed a lot to the prediction of fine-grained emotions such as “sentimental”, “disappointed”, and “hopeful”. “Light/dark” contributes more to the prediction of “depressed”.

**Figure 9:**
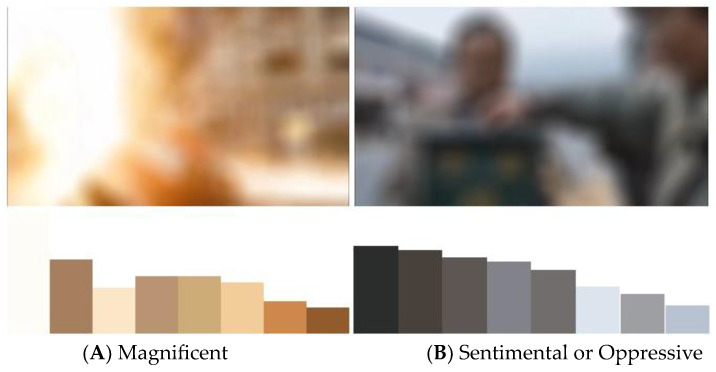
The two examples of video samples with their multicolor schemes using K-means clustering [19].

Combined with Figure 9, we can further explain the mechanism of emotion excitation by color perception. Taking “gaudy/plain” as an example, the score of the perceptual feature of “gaudy/plain” in Figure 9A is 1.8, indicating that it is relatively gaudy, and the emotion with the highest score is “magnificent”, which again proves that “gaudy” can stimulate the emotion of “magnificent”, and its score of “magnificent” is 4.1. Similarly, the score of “gaudy/plain” in Figure 9B is 4.5, indicating that it is relatively plain, which can stimulate the emotions of “sentimental”, “oppressive”, “lonely”, and “disappointed”. Therefore, we can give an explanation that Figure 9A has higher saturation, brightness, and contrast, resulting in a more vibrant and visually striking image, evoking a sense of magnificence and richness, which tends to elicit more positive emotions such as “magnificent” and “happy”. In contrast, Figure 9B has lower brightness, contrast, and saturation, with low-brightness and -saturation colors often considered as heavy and depressing, more likely to evoke negative emotions such as “lonely”.

To further verify the important role of perceptual features, we only use the top 24 important dimensional features as input features for prediction. The results are shown in Table 10.

The top 24 dimensional features of each emotion word roughly contain three perceptual features, which are considered to be the three perceptual features that can most arouse such emotion words, and the importance value of the 24th feature is about 0.01 on average, which is very small. So, we believe that the features after the 24th feature have little effect on emotion elicitation. So, the first 24 dimensions were chosen. The experimental results are shown in Table 10.

The results using only the first 24 dimensional features as input are better than those using the full number of dimensions. The possible reason is that among the first 24 dimensional features, the proportion of color perceptual features related to emotion is relatively increased, so as to capture the association between perceptual features and emotion more effectively.

In addition, using all features may bring noise and lead to over-fitting, so using all features may not achieve the best sentiment prediction effect. In contrast, by selecting important features and incorporating them into the modeling process, the fine-grained emotion prediction in the video can be more accurate and effective. This experimental result provides practical guidance on the importance of feature selection in emotion prediction tasks.

## 5. Conclusions

This study investigates the influence of color perceptual features on the fine-grained emotion prediction of video clips. Finally, a fine-grained emotion prediction model is constructed by using the random forest algorithm, which explains the correlation between the color visual perception of color and fine-grained emotion from the perspective of visual features.

Specifically speaking, the color perceptual features consist of “dynamic/static”, “gaudy/plain”, “cool/warm”, “light/dark”, “light/heavy”, “soft/hard”, “transparent/turbid”, “far/near”, and “swell/shrink”. The fine-grained emotion space consists of 16 sentimental words: “magnificent”, “lonely”, “happy”, “romantic”, “dreamy”, “relaxed”, “fresh”, “sentimental”, “disappointed”, “warm”, “cozy”, “hopeful”, “depressed”, “oppressive”, “sunny”, and “anxious”. The main contributions are as follows:

- A detailed analysis of the correlation between visual perceptual features of color and fine-grained emotion words was performed. In terms of color perception features, among all perception features, each feature has a large difference in its correlation with emotion, indicating that in each emotion dimension, the influence of each color perceptual feature is different. Specifically, “gaudy/plain” has the highest correlation with “magnificent” and “swell/shrink” has the lowest correlation with “disappointed”.
- The random forest algorithm was adopted to establish a fine-grained emotion prediction model based on color perception. The mathematical model performed well on our film and TV video dataset, indicating that our regression model can predict fine-grained emotion value reasonably well based on color perception features.
- Based on the research mentioned above, we analyzed the factors that affect fine-grained emotion prediction at the visual feature level of color. This study compared the importance of different perceptual quantities for each emotion dimension, explaining the mechanism of action between color perception and fine-grained emotion. The first 24 important features were selected to predict emotion, which more effectively captures the correlation between perceptual features and emotion, and proves the significant influence of perceptual features on emotion arousal.

There should be cultural differences in color–emotion associations, and there might also be differences in sex and age. However, this study expected to explore the general association between color perceptual features and fine-grained emotion, thus avoiding the influence of differences in age, gender, and culture on emotion prediction. In the following work, in order to improve the reliability and generality of the model, we will expand the scale of the datasets, increase the number of subjects, and improve the algorithm of the model to further clarify the association between video color and emotion.

## Figures and Tables

**Table 1:** The 16-dimensional fine-grained emotion space.

No.	Chinese	English	Description
1	大气	Magnificent	Very good, beautiful, or deserving to be admired
2	孤独	Lonely	Unhappy because you are not with other people
3	快乐	Happy	Feeling, showing, or causing pleasure or satisfaction
4	浪漫	Romantic	Exciting and mysterious and having a strong effect on your emotions
5	梦幻	Dreamy	Seeming to be in a dream
6	轻松	Relaxed	Feeling happy and comfortable because nothing is worrying you
7	清新	Fresh	New or different
8	伤感	Sentimental	Too strongly influenced by emotional feelings
9	失落	Disappointed	Feelings related to loss, no hope, or bad mood
10	温暖	Warm	Feeling warm and comforted
11	温馨	Cozy	Warm and aromatic
12	希望	Hopeful	Thinking of achieving a certain goal or situation
13	消沉	Depressed	Unhappy and without hope
14	压抑	Oppressive	To control emotions, power, etc., not to be fully revealed or exerted
15	阳光	Sunny	Positive, optimistic, and cheerful
16	忧虑	Anxious	Being distressed by what has happened or by worrying about what will happen in the future

**Table 2:** The result of the reliability analysis.

No.	Descriptive Variable	Cronbach’s Alpha
**1**	Magnificent	0.922
**2**	Lonely	0.739
**3**	Happy	0.954
**4**	Romantic	0.969
**5**	Dreamy	0.984
**6**	Relaxed	0.967
**7**	Fresh	0.974
**8**	Sentimental	0.702
**9**	Disappointed	0.818
**10**	Warm	0.958
**11**	Cozy	0.962
**12**	Hopeful	0.961
**13**	Depressed	0.847
**14**	Oppressive	0.819
**15**	Sunny	0.952
**16**	Anxious	0.881

**Table 3:** The nine multicolor perceptual features.

No.	Chinese	English	Description
1	动–静	Dynamic–static, DS	Dynamic: change the original position or statement; Static: keep the original position or statement.
2	华丽–朴素	Gaudy–plain, GP	Gaudy: beautiful and glorious; Plain: (color, style, etc.) not rich or gaudy.
3	冷–暖	Cool–warm, CW	Cool: (feel) lower temperature; Warm: (feel) higher temperature.
4	明–暗	Light–dark, LD	Light: bright and full of light; Dark: lack of light as opposed to light.
5	轻–重	Light–heavy, LH	Light: bright and full of light; Dark: lack of light as opposed to light.
6	软–硬	Soft–hard, SH	Soft: loose internal structure, easy to deform; Hard: tight internal structure, difficult to deform.
7	透明–浑浊	Transparent–turbid, TT	Soft: loose internal structure, easy to deform; Hard: tight internal structure, difficult to deform.
8	远–近	Far–near, FN	Far: long space distance; Near: short space distance.
9	胀–缩	Swell–shrink, SS	Swell: from small to large or from short to long; Shrink: from large to small or from long to short.

Note: The data cite from the research of Wang et al. [1].

**Table 4:** The results of the reliability analysis.

No.	Descriptive Variable	Cronbach’s Alpha
**1**	Dynamic–static, DS	0.943
**2**	Gaudy–plain, GP	0.942
**3**	Cool–warm, CW	0.730
**4**	Light–dark, LD	0.850
**5**	Light–heavy, LH	0.793
**6**	Soft–hard, SH	0.793
**7**	Transparent–turbid, TT	0.841
**8**	Far–near, FN	0.855
**9**	Swell–shrink, SS	0.806

**Table 5:** Pearson correlation coefficients between different emotion words and perceived quantities.

Correlation Coefficient	DS	GP	CW	LD	LH	SH	TT	FN	SS
Magnificent	−0.412	−0.785	0.187	−0.169	0.060	0.020	0.198	0.042	−0.053
Lonely	0.224	0.367	−0.179	0.339	0.168	0.253	0.172	−0.206	−0.043
Happy	−0.051	−0.129	0.073	−0.233	−0.225	−0.299	−0.266	0.046	0.177
Romantic	0.148	−0.167	0.027	−0.019	−0.281	−0.420	−0.266	0.107	0.285
Dreamy	0.031	−0.038	0.179	0.092	0.000	−0.143	−0.112	0.061	−0.054
Relaxed	−0.010	−0.024	−0.090	−0.153	−0.283	−0.278	−0.342	−0.028	0.310
Fresh	0.118	−0.029	−0.054	−0.121	−0.353	−0.355	−0.333	−0.125	0.321
Sentimental	0.167	0.000	0.037	0.218	0.212	0.073	0.148	−0.420	0.027
Disappointed	0.119	0.136	0.018	0.190	0.160	0.120	0.058	−0.396	0.009
Warm	0.124	−0.115	0.175	−0.139	−0.209	−0.352	−0.163	0.072	0.150
Cozy	0.120	−0.079	0.115	−0.133	−0.227	−0.345	−0.194	−0.010	0.137
Hopeful	−0.141	−0.224	0.068	−0.207	−0.152	−0.178	−0.094	0.318	0.076
Depressed	0.111	0.150	−0.021	0.236	0.264	0.208	0.186	−0.282	−0.024
Oppressive	0.002	0.075	0.043	0.179	0.301	0.283	0.269	−0.196	−0.188
Sunny	−0.120	−0.131	0.067	−0.266	−0.196	−0.233	−0.211	0.075	0.168
Anxious	0.133	0.219	−0.003	0.188	0.338	0.219	0.144	−0.257	−0.066

Note: Green indicates that there is a weak correlation. Yellow indicates that there is a strong correlation.

**Table 6:** Comparison of results initially and blurred video features.

Feature Source	MAE	MSE	RMSE
Original video	0.198	0.061	0.245
Blurred video	0.195	0.058	0.238

**Table 7:** The average results of RF, SVR, and MLP.

Model	MAE	MSE	RMSE
RF	0.195	0.058	0.238
SVR	0.193	0.061	0.243
MLP	0.247	0.096	0.302

**Table 8:** The average results of RF with different feature combinations.

Feature	MAE	MSE	RMSE
Physical features + 9 dimensional perception features	0.195	0.058	0.238
Physical features	0.203	0.064	0.249
Physical features + DS	0.201	0.063	0.247
Physical features + GP	0.201	0.062	0.246
Physical features + CW	0.201	0.063	0.248
Physical features + LD	0.203	0.063	0.248
Physical features + LH	0.200	0.063	0.247
Physical features + SH	0.198	0.061	0.243
Physical features + TT	0.201	0.063	0.248
Physical features + FN	0.199	0.062	0.245
Physical features + SS	0.202	0.064	0.249

**Table 9:** The top-ranked perceptual features and the importance value of the perceptual feature corresponding to each emotion word.

Emotion Words	Perception Feature	Importance Value
Magnificent	Gaudy–plain, GP	0.631
Lonely	Gaudy–plain, GP	0.062
Happy	Soft–hard, SH	0.032
Romantic	Far–near, FN	0.042
Dreamy	Soft–hard, SH	0.064
Relaxed	Swell–shrink, SS	0.026
Fresh	Light–heavy, LH	0.044
Sentimental	Far–near, FN	0.081
Disappointed	Far–near, FN	0.047
Warm	Soft–hard, SH	0.040
Cozy	Soft–hard, SH	0.037
Hopeful	Soft–hard, SH	0.028
Depressed	Light–dark, LD	0.024
Oppressive	Light–heavy, LH	0.016
Sunny	Soft–hard, SH	0.015
Anxious	Light–heavy, LH	0.037

**Table 10:** The results of RF with the top 24 important dimensional features.

Emotion Words	MAE	MSE	RMSE
Average result	0.185	0.053	0.227
Magnificent	0.187	0.054	0.231
Lonely	0.214	0.030	0.268
Happy	0.146	0.030	0.173
Romantic	0.217	0.067	0.259
Dreamy	0.168	0.037	0.194
Relaxed	0.198	0.051	0.225
Fresh	0.170	0.039	0.198
Sentimental	0.162	0.037	0.193
Disappointed	0.271	0.093	0.305
Warm	0.189	0.048	0.220
Cozy	0.207	0.061	0.248
Hopeful	0.151	0.034	0.185
Depressed	0.195	0.088	0.297
Oppressive	0.182	0.060	0.245
Sunny	0.135	0.028	0.167
Anxious	0.176	0.053	0.230

## Data Availability

The data that support the findings of this study are available from the corresponding author upon reasonable request. The data are not publicly available due to privacy or ethical restrictions.
